# MiR-182-5p and miR-375-3p Have Higher Performance Than PSA in Discriminating Prostate Cancer from Benign Prostate Hyperplasia

**DOI:** 10.3390/cancers13092068

**Published:** 2021-04-25

**Authors:** Irena Abramovic, Borna Vrhovec, Lucija Skara, Alen Vrtaric, Nora Nikolac Gabaj, Tomislav Kulis, Goran Stimac, Dejan Ljiljak, Boris Ruzic, Zeljko Kastelan, Bozo Kruslin, Floriana Bulic-Jakus, Monika Ulamec, Ana Katusic-Bojanac, Nino Sincic

**Affiliations:** 1 Department of Medical Biology, University of Zagreb School of Medicine, 10000 Zagreb, Croatia; irena.abramovic@mef.hr (I.A.); lucija.skara@mef.hr (L.S.); floriana.bulic@mef.hr (F.B.-J.); ana.katusic@mef.hr (A.K.-B.); 2 Group for Research on Epigenetic Biomarkers (Epimark), University of Zagreb School of Medicine, 10000 Zagreb, Croatia; tkulis@gmail.com (T.K.); goran.stimac2@zg.t-com.hr (G.S.); boris.ruzic@zg.t-com.hr (B.R.); zeljko.kastelan@gmail.com (Z.K.); monika.ulamec@gmail.com (M.U.); 3 Centre of Excellence for Reproductive and Regenerative Medicine, University of Zagreb School of Medicine, 10000 Zagreb, Croatia; alenvrtaric@gmail.com (A.V.); nora.nikolac@gmail.com (N.N.G.); bozo.kruslin@mef.hr (B.K.); 4 Department of Urology, University Clinical Hospital Center Sestre Milosrdnice, 10000 Zagreb, Croatia; borna.vrhovec@gmail.com; 5 Department of Clinical Chemistry, University Clinical Hospital Center Sestre Milosrdnice, 10000 Zagreb, Croatia; 6 Faculty of Pharmacy and Biochemistry, University of Zagreb, 10000 Zagreb, Croatia; 7 Department of Urology, University Hospital Centre Zagreb, 10000 Zagreb, Croatia; 8 Department of Gynecology and Obstetrics, University Clinical Hospital Center Sestre Milosrdnice, 10000 Zagreb, Croatia; dejan.ljiljak@kbcsm.hr; 9 Ljudevit Jurak Clinical Department of Pathology and Cytology, University Clinical Hospital Center Sestre Milosrdnice, 10000 Zagreb, Croatia; 10 Department of Pathology, School of Dental Medicine and School of Medicine, University of Zagreb, 10000 Zagreb, Croatia

**Keywords:** prostate cancer, microRNA, liquid biopsy, biomarkers, benign prostate hyperplasia, plasma, seminal fluid

## Abstract

**Simple Summary:**

Prostate cancer (PCa) is the most prevalent neoplasia among men worldwide but is commonly “mimicked” by benign prostate hyperplasia (BPH). Their discrimination by the prostate-specific antigen (PSA) is often uncertain, resulting in lengthy diagnostic protocols and recurrent tissue biopsies. The development of more appropriate biomarkers, possibly present in liquid biopsy, would significantly improve PCa and BPH patient management. To address this challenge, in this study miR-375-3p, miR-182-5p, miR-21-5p, and miR-148a-3p were analyzed by ddPCR in blood plasma and seminal plasma of patients with PCa and BPH prior to tissue biopsy. Among other findings, miR-182-5p and miR-375-3p were found to have statistically significantly higher expression in PCa patients compared to BPH in blood, with a combined specificity of 90.2% to predict positive or negative biopsy results. The data presented emphasize the great potential of miRNAs as liquid biopsy biomarkers for PCa.

**Abstract:**

Prostate cancer (PCa) is the most commonly diagnosed neoplasm among men. Since it often resembles benign prostate hyperplasia (BPH), biomarkers with a higher differential value than PSA are required. Epigenetic biomarkers in liquid biopsies, especially miRNA, could address this challenge. The absolute expression of miR-375-3p, miR-182-5p, miR-21-5p, and miR-148a-3p were quantified in blood plasma and seminal plasma of 65 PCa and 58 BPH patients by digital droplet PCR. The sensitivity and specificity of these microRNAs were determined using ROC curve analysis. The higher expression of miR-182-5p and miR-375-3p in the blood plasma of PCa patients was statistically significant as compared to BPH (*p* = 0.0363 and 0.0226, respectively). Their combination achieved a specificity of 90.2% for predicting positive or negative biopsy results, while PSA cut-off of 4 µg/L performed with only 1.7% specificity. In seminal plasma, miR-375-3p, miR-182-5p, and miR-21-5p showed a statistically significantly higher expression in PCa patients with PSA >10 µg/L compared to ones with PSA ≤10 µg/L. MiR-182-5p and miR-375-3p in blood plasma show higher performance than PSA in discriminating PCa from BPH. Seminal plasma requires further investigation as it represents an obvious source for PCa biomarker identification.

## 1. Introduction

Prostate cancer (PCa) is the most commonly diagnosed neoplasm among men in the Western world with constantly rising incidence worldwide [1]. The PCa diagnosis is confirmed by histopathological analysis of prostate biopsy cores, after a suspiciously elevated prostate-specific antigen (PSA) or positive digital rectal examination (DRE). PSA has low specificity and only in up to 30% of patients with PSA 4–10 ng/uL PCa is it confirmed by histopathology [2]. Benign prostate hyperplasia (BPH) is the most common condition that presents with high PSA levels “mimicking” PCa. Such findings often result in a tissue biopsy, which is the only clinical tool powerful enough to distinguish these diseases with different therapy protocols and outcomes. Indeed, many unnecessary biopsies are being performed causing significant stress and risk for patients, as well as a financial and logistic burden for a healthcare system. To overcome these challenges, new biomarkers with higher sensitivity and specificity are under intensive research. A special focus has been put on liquid biopsy biomarkers, especially in the blood due to their minimal invasiveness and fast real-time analysis [3,4].

Among liquid biopsy biomarkers, the most prominent ones are small non-coding RNAs (miRNAs), epigenetically active oligonucleotides that post-transcriptionally regulate gene expression [5]. Dysregulation of miRNAs was described in PCa tissue in 2006 and soon after in sera of PCa patients [6,7]. These discoveries led to a sharp increase in the number of related studies. Still, miRNAs as PCa biomarkers have many challenges that should be overcome before significant progress could be achieved. The most challenging, are the lack of widely accepted guidelines regarding sample processing and storage, experimental protocols, miRNA isolation and quantification, data analysis, and reliable miRNA for normalization [8,9]. Some of these challenges can be addressed by the use of digital droplet PCR (ddPCR). This technology allows absolute quantification overcoming the normalization issue, as well as replicate samples [10,11]. Apart from technical issues regarding miRNA quantification, most promising miRNAs were later found of low or no value for clinical use as PCa biomarkers, especially in patient’s liquid biopsies. Therefore, the identification of more specific and sensitive miRNAs in liquid biopsies as PCa biomarkers is still a challenge waiting to be addressed. Contrary to the standard relationship with blood and urine in comparison to other tissues, prostate tissue is strongly related to seminal fluid contributing to its volume by 40% by secreting prostatic fluid. It is reasonable to expect that PCa-related miRNAs could be detected easier in seminal plasma than in blood or urine [12]. Even so, seminal fluid is a source of miRNAs in PCa patients that is the least researched, with only several papers published [13,14,15,16].

The present study aimed to investigate the possible clinical value of the miR-375-3p, miR-182-5p, miR-148a-3p, and miR-21-5p as discriminatory biomarkers between PCa and BPH patients when analyzed in patients’ blood plasma and seminal plasma using ddPCR technology. In fact, miR-375-3p and miR-21-5p have already been under some interest as cancer biomarkers, but there are almost no data on seminal plasma expression in PCa patients. MiR-148a-3p and miR-182-5p in liquid biopsies as cancer biomarkers are still under research and greatly underestimated despite confirmed overexpression in PCa tissue [8]. Truly, these miRNAs represent a valuable target for research on PCa biomarkers especially in discriminating this malignancy from benign changes in prostate tissue like BHP.

## 2. Materials and Methods

### 2.1. Study Population

In this prospective study, 123 patients scheduled for prostate biopsy due to clinical suspicion of PCa were included from October 2018 to June 2020, at the University Hospital Center Sestre Milosrdnice and University Hospital Center, Zagreb. Based on histopathological analysis of prostate core biopsies performed by a pathologist, patients were divided into two groups: 65 in PCa and 58 in the BPH group. For each patient PSA level in serum, Gleason Score (GS) and WHO 2016/ISUP (International Society of Urological Pathology) grade groups data were collected from routine patient medical documentation.

For some patients, this was a repeated biopsy, after previously negative results for PCa. Still, no patient was subjected to prostate biopsy or DRE at least six months prior to blood and semen sampling for this study, so as to avoid possible interference of recent prostate tissue manipulation on miRNA expression in liquid biopsy.

The study was performed under the approval of the Ethical Committees of the University Hospital Center Sestre Milosrdnice, University Hospital Center, Zagreb, and University of Zagreb School of Medicine. The research was conducted in accordance with the Declaration of Helsinki (2011), and written informed consent was obtained from every patient before enrolment into the study. All patients were thoroughly informed about the details of the study and were able to withdraw from the study of their own free will at any time. Patients’ personal data were collected, coded, stored, analyzed, and presented following effective national legislation.

### 2.2. Sample Collection

Peripheral venous blood samples were collected before biopsy in 6 mL EDTA-containing BD™ Vacutainer^®^ Blood Collection Tubes (Becton Dickinson, NJ, USA) and immediately processed into plasma by dual centrifugation (at 1400× *g* and 4500× *g*, both for 10 min at room temperature). All patients included in the study provided blood samples.

Semen samples were obtained by masturbation after 3–5 days of sexual abstinence, one day before the biopsy, and processed after 30–60 min liquefaction at room temperature into seminal plasma by dual centrifugation (at 400× *g* and 12000× *g*, both for 10 min at room temperature). All fractions were subsequently stored at −80 °C until analysis. All further analysis was performed on the Department of Medical Biology at the University of Zagreb School of Medicine.

### 2.3. MiRNA Analysis

Total RNA was isolated from 200 µL of blood and seminal plasma using miRNeasy Serum/Plasma Advanced Kit (Qiagen, Hilden, Germany), according to the instructions in the manual. We applied TaqMan™ MicroRNA Reverse Transcription Kit (Applied Biosystems, Foster City, CA, USA) to conduct reverse transcription of all miRNA in the sample into cDNA using universal primer, according to the manufacturer’s instructions. A fixed volume of the eluted RNA sample was used as input, in order to normalize the sample-to-sample variability in miRNA content. The cDNA was stored at −20 °C and further analyzed within a month.

Because of ddPCR high sensitivity, cDNA samples were diluted in Mili-Q^®^ water before miRNA quantification according to our optimized protocol. When isolated from blood plasma miR-375-3p and miR-182-5p samples were diluted to 1:50, miR-148a-3p to 1:250, and miR-21-5p to 1:10.000. When isolated from seminal plasma, all miRNA samples were diluted 1:100. Total miR-375-3p, miR-182-5p, miR-148a-3p, and miR-21-3p in every sample was quantified using the digital droplet PCR (ddPCR) system (Bio-Rad Laboratories, Hercules, CA, USA). Briefly, 2 µL of the diluted cDNA were added to the 20 µL PCR reaction mixture containing 10 µL of ddPCR Supermix for Probes (Bio-Rad), 1 µL of TaqMan primer/probe mix (Applied Biosystems, Foster City, CA, USA), and 7 µL of RNase-free H2O. Each ddPCR assay mixture was loaded into the disposable DG8 Cartridge (Bio-Rad, Hercules, CA, USA) located in a cartridge holder (BioRad). Seventy (70) µL of Droplet Generation Oil for Probes (Bio-Rad) was loaded into every well. The cartridge was covered with the DG8 Gasket (Bio-Rad) and placed inside the QX200 Droplet Generator (Bio-Rad) where every sample was partitioned into 20,000 nanoliter-sized droplets, with target and background cDNA being randomly distributed into the droplets. After droplet generation, droplets were transferred to a ddPCR plate (BioRad), heat-sealed with a pierceable aluminum foil (Bio-Rad) in the PX1 PCR Plate Sealer (Bio-Rad). PCR amplification was carried out on the CFX96 Deep Well PCR thermal cycler (BioRad). Thermal cycling conditions were 95 °C for 10 min, 50 cycles of 94 °C for 30 s and 57.5 °C for 1 min, 98 °C for 10 min and infinite holding at 4 °C; with temperature ramp rate set to 2 °C/s for every step during the cycling. No template control (NTC) was included in every assay. QX200 Droplet Reader (Bio-rad, Hercules, CA, USA) was applied for analyzing every droplet individually, using a two-color detection system. PCR-positive (did contain target) and PCR-negative (did not contain target) droplets were counted, respectively, to provide absolute quantification of target DNA using QuantaSoft software. Quantification was performed assuming a Poisson distribution. The results were converted in the number of copies present in the starting sample by multiplying concentration values given by the software with the reaction volume, divided with the starting volume of cDNA, and multiplied by the corresponding dilution factor to obtain the total copy number per µL of sample plasma. The data were acquired using the 1-dimensional or 2-dimensional based plotting systems as recommended by the manufacturer. Thresholds were set by excluding only the true negative population, according to the no template control (NTC) which was included in each assay [17].

### 2.4. Statistical Analysis

Statistical analysis was performed using GraphPad Prism software (Windows 5.00, GraphPad Software, San Diego, CA, USA). A non-parametric test, Mann–Whitney sum test, was used to compare miR-375-3p, miR-182-5p, miR-148a-3p, and miR-21-3p expression levels between PCa and BPH groups. Spearman′s rank correlation was applied to correlate miR-375-3p, miR-182-5p, miR-148a-3p, and miR-21-3p expression levels with clinicopathological parameters. The receiver operating characteristic (ROC) curve was constructed and the area under the curve (AUC) was calculated to determine the potential of miRNAs to discriminate between PCa and BPH samples. The highest Youden’s J index (sum of sensitivity and specificity—1) was used to set up corresponding cut-off value with maximized sensitivity and specificity for each ROC analysis [18]. Multiple logistic regression analysis was performed to assess the association of target miRNAs′ expression with PCa and BPH. A *p* value < 0.05 (two-tailed) was considered statistically significant.

## 3. Results

### 3.1. Patient’s Characteristics

From a total of 123 enrolled patients, 65 were diagnosed with PCa and 58 with BPH, forming corresponding experimental groups. No difference in age (*p* = 0.2298) or pre-biopsy serum PSA level (*p* = 0.9399) was found. Of all cancer patients, most had a GS of 7, of which 75% had GS 3 + 4 belonging to WHO 2016/ISUP grade 2 (Table 1).

### 3.2. Blood and Seminal Plasma Expression of miRNAs

**Figure 1:**
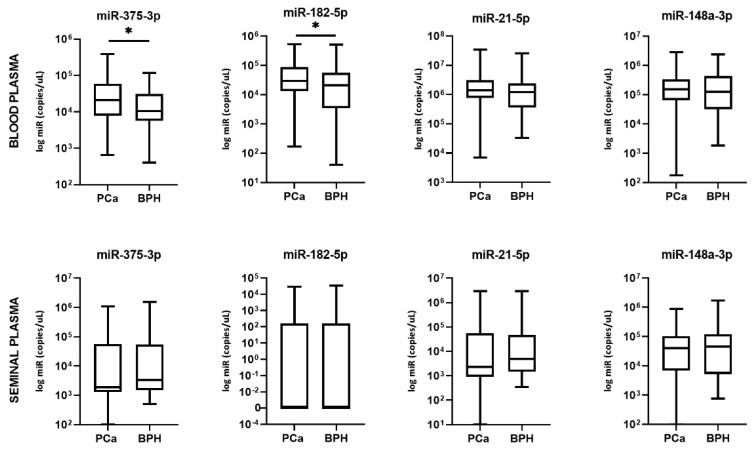
Blood and seminal plasma miRNA levels in PCa patients compared to BPH patients. Significant differences between groups are indicated: * *p* < 0.05.

Absolute quantification analysis revealed that miR-182-5p in blood plasma had significantly higher expression in PCa patients compared to BPH (*p* = 0.0363) (Figure 1). Even higher significance was found for the expression level of miR-375-3p (*p* = 0.0226). The difference in expression of miR-21-5p and miR-148a-3p showed no statistical significance in discriminating PCa from BPH patients.

**Figure 2:**
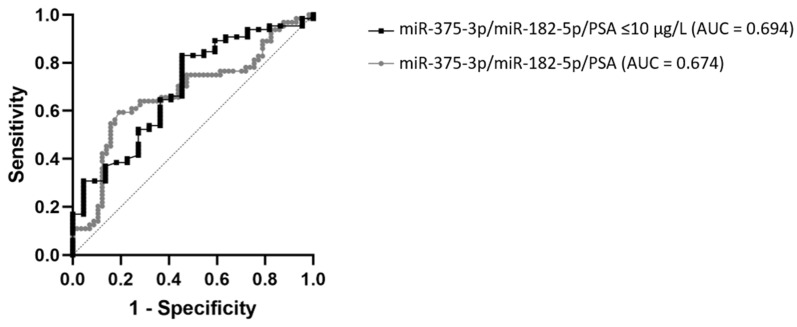
ROC curve analysis of the combinations miR-375-3p/miR-182-5p/PSA and miR-375-3p/miR-182-5p/PSA using sub-cohort of PCa patients with serum PSA levels ≤10 µg/L. is a figure.

ROC curve analysis of miR-182-5p expression showed 64.6% sensitivity and 61.4% specificity in discriminating between PCa and BPH patients, corresponding to an AUC of 0.610 (95% CI: 0.508–0.712, *p* = 0.0365). For miR-375-3p 40% sensitivity and 80.7% specificity was detected, corresponding to an AUC of 0.620 (95% CI: 0.521–0.719, *p* = 0.0229). Both miRNAs displayed much better diagnostic performance than PSA (AUC = 0.504, 95% CI: 0.401–0.608, *p* = 0.939), which showed 95.3% sensitivity and 1.7% specificity at its cut-off of 4 µg/L. By using multiple logistic regression and combining miR-375-3p, miR-182-5p, and PSA, the ROC curve was constructed for PSA, using cut-off values for miRNAs determined based on the Youden index. This analysis showed an improved AUC of 0.674 (95% CI: 0.576–0.772, *p* = 0.001). When applying it only on patients with PSA ≤10 µg/L, the ROC curve was further improved to AUC 0.694 (95% CI: 0.567–0.821, *p* = 0.0068) (Figure 2). The comparison of these two ROC curves did not show a significant difference between AUC values (*p* = 0.8089). When sum scores were generated according to if miRNA expression levels predicted a positive (1) or negative (0) biopsy result, specificity reached 90.2%, with sensitivity at 39.1% (AUC = 0.653, 95% CI: 0.538–0.768, *p* = 0.014) for sum score >1.5, meaning both miRNA levels were above the cut-off value. When either one of miRNA was above the cut-off value, specificity was 53.7% and sensitivity 65.2%.

There was no statistically significant difference between PCa and BPH groups regarding seminal plasma expression of miR-375-3p, miR-182-5p, miR-21-5p, and miR-148a-3p (*p* = 0.2038, 0.9562, 0.5186, and 0.8582, respectively) (Figure 1). Expression of miR-182-5p was not detected at all in more than half of the samples in both patient groups.

### 3.3. Association of miRNAs with Clinicopathological Variables of PCa Patients

In order to investigate the association of miRNA with PCa aggressiveness, we compared miRNA expression levels in PCa patients with different WHO 2016/ISUP grade groups, Iow/high WHO 2016/ISUP grade group (≤2 vs ≥3), low/high biopsy GS group (=6 vs ≥7), and low/high PSA levels (≤10 vs >10 µg/L).

**Figure 3:**
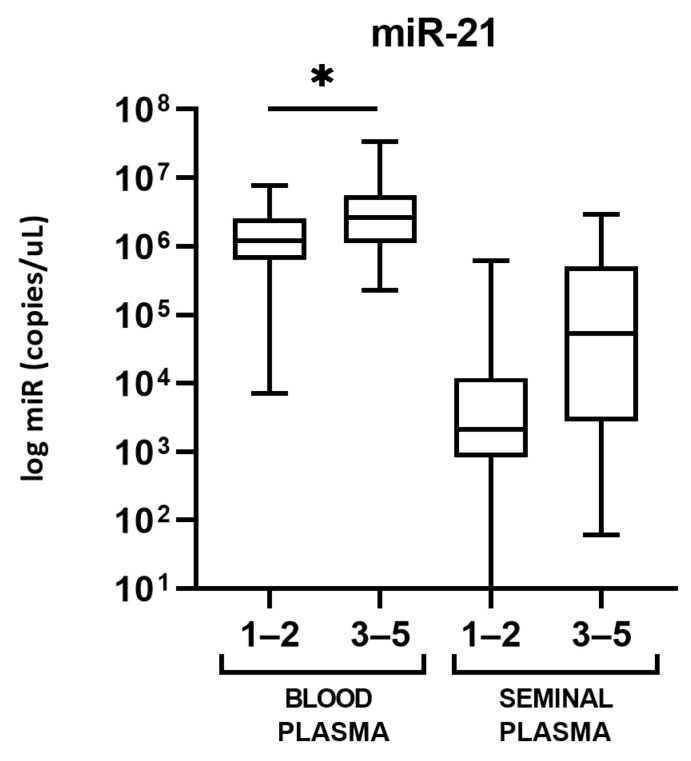
Blood and seminal plasma levels of miR-21-5p in PCa patients with WHO 2016/ISUP grade groups 1–2 compared to WHO 2016/ISUP grade groups 3–5. Significant differences between grade groups are indicated: * *p* < 0.05 and seminal plasma miRNA levels in PCa patients compared to BPH patients. Significant differences between groups are indicated: * *p* < 0.05.

In blood plasma, miR-21 expression was statistically higher in PCa patients belonging to WHO 2016/ISUP grade groups 3 to 5, as compared to the patients with WHO 2016/ISUP grade groups 1 and 2 (*p* = 0.0265). There is a tendency toward a higher expression of miR-21 in seminal plasma in high-grade PCa as well, but it was not found as statistically significant (*p* = 0.0686) (Figure 3). No statistically significant difference was found between WHO 2016/ISUP grade groups as well, neither in blood nor in seminal plasma. There was no statistically significant difference between PCa patients with GS 6 compared to GS 7 or higher for none of the miRNA in neither blood nor seminal plasma.

**Figure 4:**
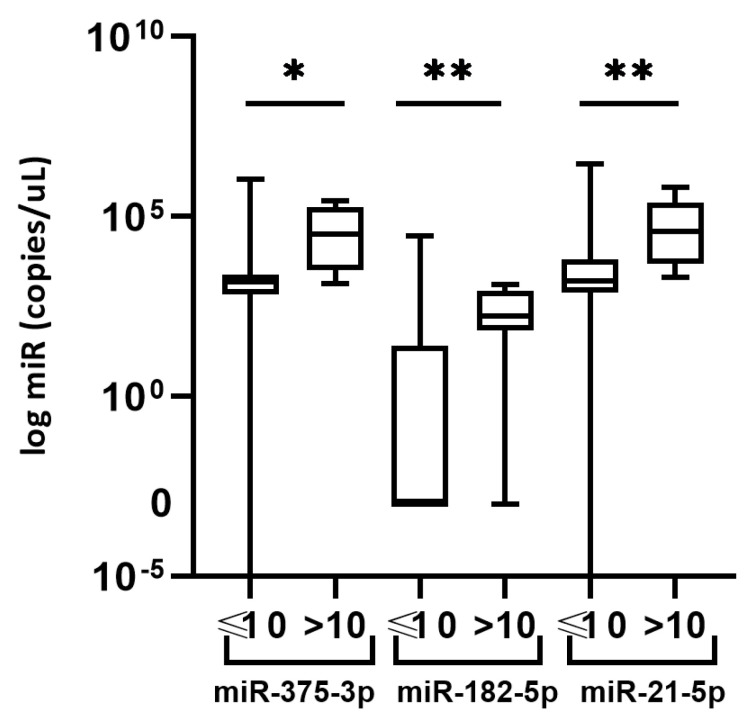
Seminal plasma expression of miR-375-3p, miR-182-5p, and miR-21-5p in PCa patients with serum PSA levels ≤10 vs >10 µg/L. Significant differences between groups are indicated: * *p* < 0.05; ** *p* < 0.01.

Regarding low/high PSA levels, we observed a statistically significant higher expression of miR-375-3p, miR-182-5p, and miR-21-5p (*p* = 0.0147, 0.0040, and 0.0082, respectively) in seminal plasma of PCa patients with PSA levels >10 µg/L, as compared to the PCa patients with PSA levels ≤10 µg/L (Figure 4).

The correlation of miRNA expression with clinicopathological parameters (age, serum PSA levels, GS, and WHO 2016/ISUP grade group) was examined in PCa patients for every miRNA in both blood and seminal plasma samples. Expression of all analyzed miRNAs appears stable across the age since no correlation with age was found. PSA levels positively correlated with seminal plasma expression of miR-375-3p, miR-182-5p, and miR-21-5p (Spearman correlation coefficients: r = 0.4238 (*p* = 0.1250), 0.4404 (*p* = 0.0091), and 0.5275 (*p* = 0.0013)). In blood plasma, miR-21 showed statistically significant correlation with WHO 2016/ISUP grade group (Spearman correlation coefficient r = 0.257 (*p* = 0.039)), while miR-375 with both GS and WHO 2016/ISUP grade group (r = 0.299 (*p* = 0.0154) and r = 0.254 (*p* = 0.041), respectively).

### 3.4. MiRNAs Co-Expression

Concerning mutual miRNA correlation, the expression levels of all miRNAs showed a statistically significant positive correlation with each other in blood plasma (Supplementary Table S1). The strongest correlation was observed between levels of miR-375-3p/miR-182-5p (r = 0.6346, *p* < 0.0001) and miR-182-5p/miR-148a-3p (r = 0.6031, *p* < 0.0001). Likewise, all miRNAs mutually positively correlated in seminal plasma as well, apart from miR-182-5p/miR-148a-3p (Supplementary Table S1). Moreover, a very strong correlation was found for miR-375-3p/miR-21-5p (r = 0.7933, *p* < 0.0001) and miR-182-5p/miR-21-5p (r = 0.8496, *p* < 0.0001). Statistically significant correlation between blood and seminal plasma was found for miR-21-5p (r = 0.3710, *p* = 0.0282).

## 4. Discussion

In recent years, the research has been focused on liquid biopsies as a source of potential biomarkers for improving PCa diagnosis, prognosis, and patient management. The expression of miRNAs in serum, plasma, or urine is being eagerly studied for this purpose [19,20,21]. Still, there are almost no published studies on seminal plasma as a potential source of miRNA as a PCa biomarker. In the present study, we investigated the expression of cancer-related miR-375-3p, miR-182-5p, miR-21-5p, and miR-148a-3p in blood plasma and seminal plasma of men undergoing biopsy due to the elevated PSA levels and suspicion on PCa. According to the histopathological examination, these patients were later distinguished as PCa and BPH patients. The obtained ddPCR data showed statistically significantly higher expression of miR-375-3p and miR-182-5p in blood plasma in men with PCa, as compared to men with BPH. These two miRNAs showed significantly better diagnostic characteristics than PSA, in terms of specificity and sensitivity, as we show in the Results and as was indicated by other researchers [22]. We further assessed their clinical value by constructing the ROC curve and combining its expression with PSA levels, which resulted in AUC = 0.694. Moreover, we reported great specificity of 90.2% for a combination of these miRNA cutoffs when predicting the biopsy result as positive or negative for PCa.

MiR-182-5p has been explored in plasma of PCa patients in only one study so far by Bidarra and Constancio et al., where elevated levels in PCa were reported as well [23]. They described a somewhat higher specificity of miR-182-5p (77.9%), but lower sensitivity (47.6%) [23]. However, we compared miR-182-5p expression in PCa with BPH patients, while they compared it with asymptomatic controls. The greatest challenge in clinical practice is to differentiate PCa and BPH since they differ greatly in treatment and management [3]. Therefore, study design where PCa is contrasted to BPH seems like a direction that has the most value for translational research. There is also a difference in plasma processing conditions since we used platelet-poor plasma obtained by two-step centrifugation, as opposed to one-step centrifugation [23]. It has been shown that the measurement of miRNAs is critically dependent on the removal of residual platelets before freezing plasma samples [24,25,26]. Eventually, the potential of miR-182-5p has been shown in PCa tissue [27], contributing to the understanding of the miR-182-5p role in recognized drivers of PCa–Wnt and PI3K pathways [28] and representing a possibly attractive miRNA for research and diagnostic use.

Regarding miR-375-3p, our results are in concordance with the majority of the previously published studies where plasma miR-375-3p expression was compared in PCa and BPH patients [29,30,31]. However, plasma downregulation of miR-375 in PCa compared to BPH was also observed [32], and no difference in expression was observed by Bidarra and Constancio et al. [23]. MiR-375 expression was often associated with PCa progression [27], but we have not found the difference in its expression between high and low-grade PCa when different GS or WHO 2016/ISUP grade groups were compared. However, the majority of our PCa patients were diagnosed with lower-stage PCa, which may have influenced the results.

A great potential of miR-182-5p and miR-375-3p lays in the fact that their combined levels in blood plasma could predict positive or negative biopsy results for PCa with 90.2% specificity, as we have shown.

Regarding the two other miRNAs we studied, miR-21-5p has not shown the difference in plasma expression between studied groups, despite being recognized as onco-miRNA involved in a positive expression feedback loop, together with an androgen-receptor [33]. Many studies showed its overexpression in PCa, but opposing data have been published as well [8]. However, it has been repeatedly correlated with tumor progression, especially in tumor tissue [27], corresponding with our findings which show higher expression in WHO 2016/ISUP grade groups 3–5 compared to groups 1–2 (Figure 3). The WHO 2016/ISUP grade group system was introduced to allow better prediction of patient outcome, by better discriminating GS 3 + 4 = 7 (WHO 2016/ISUP grade group 2) and GS 4 + 3 = 7 (WHO 2016/ISUP grade group 3), which differ in aggressiveness and prognosis [34]. That could explain why we found no difference in miR-21-5p expression between PCa patients with GS 6 compared to patients with GS 7.

Recently, Pastor-Navarro and García-Flores et al. proposed using a panel of four miRNAs for serum PCa diagnosis, including miR-375-3p, miR-182-5p, and miR-21-5p [35]. They showed that these miRNAs have great potential for PCa diagnostics, regarding their AUC values [35]. Again, our findings encourage these conclusions, showing that miR-182-5p and miR-375-3p deserve and require focus in further research.

MiR-148-3p has only recently started to be researched as a biomarker in liquid biopsy, after being repeatedly shown to be dysregulated in PCa tissue [8]. From studies published so far, two include using plasma as a sample and one each using sera and whole blood. They all showed overexpression in PCa compared to healthy controls [36,37,38,39]. These studies included healthy men as controls, which might be a reason for different findings in our study. Again, it is of great interest to use BPH patients as controls in PCa research since it reflects “real-life” challenges, as well as clinical needs of discerning between those two conditions. Interestingly, miR-148a-3p has been described as a miRNA with a dual role in PCa pathogenesis, meaning facilitating both PCa proliferation, as well as impairing drug-resistance in castrate-resistant PCa [40].

Seminal fluid has been proposed as a source of PCa biomarkers, especially after the differences in its components among PCa patients compared to age-matched healthy individuals have been recognized [12,41]. However, the field lacks studies in which seminal fluid is used for the investigation of its potential to discriminate PCa and BPH. To our knowledge, our study is the first one up to date where miRNA levels were analyzed in both blood and seminal plasma of the same PCa patients. Furthermore, this is the study that included the highest number of participants for research on miRNA expression in seminal plasma, as well as the largest one where BPH patients were used as controls. A similar study design was used by Barceló et al. in a significantly smaller patient subset [13,14]. In seminal fluid, we report finding no differences in the expression of miR-375-3p, miR-182-5p, miR-21-5p, and miR-148a-3p in PCa compared to BPH patients. Certainly, not observing any differences does not make the ground for eliminating miRNAs in seminal plasma as potential PCa biomarkers since other prospective miRNAs could be explored. Nonetheless, we report significantly higher levels of miR-375-3p, miR-182-5p, and miR-21-5p in PCa patients with PSA levels >10 µg/L compared to ones with PSA ≤10 µg/L. That goes in favor of data reporting their role in PCa progression [27,42,43,44].

Selth et al. were the first ones who assessed miRNA expression in the seminal fluid of PCa patients, showing higher expression compared to cancer-free controls with elevated PSA [16]. Among PCa-overexpressed miRNAs they studied, miR-375-3p was also included, but in a somewhat small validation group. Roberts et al. also looked into seminal miR-375-3p which performed with AUC 0.758 as PCa discriminating biomarker [15]. However, both of these analyses were performed in a non-sperm seminal fluid cellular fraction [16], rather than in a cell-free seminal plasma fraction.

In the aforementioned study, Barceló et al. managed to elevate specificity up to 91.7% and sensitivity up to 42.9% when combining several miRNAs originating from exosomes with PSA levels to discriminate between PCa and BPH [14], similar to what was performed in this study. Again, these valuable results should be compared with caution and hesitation, since it has not been shown that different seminal fractions can be compared. For instance, it has been shown that in plasma of PCa patients miRNA profiles from exosomes and whole plasma differ greatly [30], as was shown in other liquids and conditions [8]. Another study published by the same research group investigated miRNA expression in seminal plasma in PCa comparing it with BPH [13], with a focus on a different set of miRNAs from ours. Moreover, they proposed a combined model of three miRNAs measured in seminal plasma and PSA to predict PCa in men with elevated PSA levels [13]. Although the study was done in a small set of patients (24 with PCa and 5 with BPH), it certainly shows there is potential in seminal plasma as a source of biomarkers for PCa.

Although a respectable number of patients has been included in this research, especially regarding seminal plasma analysis for which to the best of our knowledge this is the largest study presented until now, and an important limitation of the presented results is a lack of a validation cohort. Namely, a study including a validation cohort would further test the potential of miR-375-3p and miR-182-5p translation in clinical practice as discriminatory biomarkers.

One challenge we would like to point out is the sampling of seminal fluid, which caused the patient enrolment to be challenging. Apart from the related taboo present in a population, objective challenges such as a high age of the target population prone to sexual activity disturbance and extremely low volume or compact seminal fluid, caused low compliance. This has to be taken into account in future research on seminal plasma as well as in the possible clinical development of seminal fluid biomarkers.

Another novelty of this study is using ddPCR to assess seminal miRNA levels. After investigating miRNA levels in blood plasma and seminal plasma of the same patients for the first time, we have not found any correlation in miRNA levels between these two specimen types. Still, there are no studies that clearly elucidate the physiological relationship between miRNA expression variability in different body fluids. However, a positive correlation between the expression of miRNAs in certain human tissues, including the prostate, and blood has been shown [45]. Certainly, malignant conditions lead to many changes, which could differently reflect in various body fluids, therefore representing an important area for future research on liquid biopsies.

## 5. Conclusions

This study showed that the expression of miR-182-5p and miR-375-3p in blood plasma is a superior biomarker for PCa and BPH discrimination than PSA, with potential clinical value. Further supporting their role in disease progression, miR-375-3p, miR-182-5p, and miR-21-5p demonstrated altered levels in the seminal plasma of PCa patients with different serum PSA levels. For studied miRNAs, seminal plasma did not manifest as an ideal source. However, after showing the potential of seminal fluid by other authors, further research is needed, especially due to the lack of comparable studies. Upon researching, for the first time, the expression of specific miRNAs in both blood and seminal plasma of the same patients, we highlight no correlation in the expression of these miRNAs in both specimens.

## Figures and Tables

**Table 1:** Clinicopathological details of patients included in the study.

Clinicopathological Variables	PCa (*n* = 65)	BPH (*n* = 58)
median age, years (range)	65 (40–92)	63 (47–78)
pre-biopsy PSA median, µg/L (*n*; 25–75 percentile)	7.10 (64; 5.53–10.67)	7.82 (58; 5.48–10.14)
pre-biopsy PSA levels, *n* (%)		
≤10 µg/L	46 (70.8%)	42 (72.4%)
>10 µg/L	18 (27.7%)	16 (27.6%)
unknown	1 (1.5%)	0 (0%)
GS, *n* (%)		
6	19 (29.2%)	n.a.
7	40 (61.5%)	n.a.
8	4 (6.2%)	n.a.
9	2 (3.1%)	n.a.
WHO 2016/ISUP grade		
1 (GS 3 + 3 = 6)	19 (29.2%)	n.a.
2 (GS 3 + 4 = 7)	30 (46.1%)	n.a.
3 (GS 4 + 3 = 7)	10 (15.4%)	n.a.
4 (GS 4 + 4, 3 + 5 or 5+3 = 8)	4 (6.2%)	n.a.
5 (GS 9 or 10)	2 (3.1%)	n.a.

Abbreviations: PCa, prostate cancer; BPH, benign prostatic hyperplasia; WHO, World Health Organization; ISUP, International Society of Urological Pathology; n.a., not applicable.

## Data Availability

In this study the authors have not used any data from publicly available datasets, neither have the authors generated any. Namely, as stated in Materials and Methods, according to national legislation, patients included in this study agreed that their personal data will only be used for this research, not available in public datasets. Moreover, other laboratory data are owned by institutions included in this research and therefore not available to be published in publicly available datasets.

## Supplementary Materials

The following are available online at https://www.mdpi.com/article/10.3390/cancers13092068/s1. Table S1: Correlation of clinicopathological parameters in PCa patients with miRNA levels and correlation of miRNAs in blood and seminal plasma.
