# Supplementary material for: MiR-182-5p and miR-375-3p Have Higher Performance Than PSA in Discriminating Prostate Cancer from Benign Prostate Hyperplasia
Source: Cancers (Basel). 2021 Apr 25;13(9):2068. doi: 10.3390/cancers13092068 (PMC8123314; doi:10.3390/cancers13092068)
Supplement: Supplementary file 1 [file cancers-13-02068-s001.zip › cancers-1178082-supplementary.docx]

|  | | miR-375 blood | miR-375 ejaculate | miR-182 blood | miR-182 ejaculate | miR-21 blood | miR-21 ejaculate | miR-148a blood | miR-148a ejaculate |
| --- | --- | --- | --- | --- | --- | --- | --- | --- | --- |
| age | r | 0.115 | −0.05639 | 0.1781 | -0.02733 | 0.03266 | 0.07775 | 0.996 | 0.2931 |
|  | p | 0.3617 | 0.7477 | 0.1557 | 0.8762 | 0.7962 | 0.6571 | 0.111 | 0.0875 |
| PSA | r | 0.05719 | **0.4238** | 0.01579 | **0.4404** | 0.1259 | **0.5275** | −0.07112 | 0.1692 |
|  | p | 0.6535 | **0.0125** | 0.9015 | **0.0091** | 0.3216 | **0.0013** | 0.5765 | 0.3388 |
| Gleason score | r | **0.2994** | −0.002243 | 0.09148 | 0.001687 | 0.1868 | 0.1748 | 0.09234 | 0.1052 |
|  | p | **0.0154** | 0.9898 | 0.4686 | 0.9923 | 0.1362 | 0.3153 | 0.4644 | 0.5474 |
| ISUP grade | r | **0.2542** | 0.0315 | 0.03217 | 0.153 | **0.2567** | 0.2855 | 0.003614 | 0.1082 |
|  | p | **0.041** | 0.8575 | 0.7992 | 0.3803 | **0.039** | 0.0964 | 0.9722 | 0.5361 |
| miR-375 blood | r |  | 0.1849 | **0.6346** | −0.0343 | **0.4025** | 0.1424 | **0.4581** | 0.1597 |
|  | p |  | 0.2875 | **<0.0001** | 0.8448 | **0.0009** | 0.4146 | **0.0001** | 0.3595 |
| miR-375 ejaculate | r | 0.1849 |  | −0.2486 | **0.6709** | **0.3665** | **0.7933** | −0.2396 | **0.3549** |
|  | p | 0.2875 |  | 0.1499 | **<0.0001** | **0.0304** | **<0.0001** | 0.1656 | **0.0365** |
| miR-182 blood | r | **0.6346** | −0.2486 |  | −0.2486 | **0.2935** | −0.2015 | **0.6031** | 0.2588 |
|  | p | **<0.0001** | 0.1499 |  | 0.1498 | **0.0177** | 0.2458 | **<0.0001** | 0.1332 |
| miR-182 ejaculate | r | -0.0343 | **0.6709** | −0.2486 |  | 0.2064 | **0.8496** | **−0.3856** | 0.3069 |
|  | p | 0.8448 | **<0.0001** | 0.1498 |  | 0.2342 | **<0.0001** | **0.0221** | 0.0730 |
| miR-21 blood | r | **0.4025** | 0.3665 | **0.2935** | 0.2064 |  | **0.3710** | **0.5029** | 0.0057 |
|  | p | **0.0009** | 0.0304 | **0.0177** | 0.2342 |  | **0.0282** | **<0.0001** | 0.9739 |
| miR-21 ejaculate | r | 0.1424 | **0.7933** | −0.2015 | **0.8496** | 0.3710 |  | **−0.3423** | **0.4786** |
|  | p | 0.4146 | **<0.0001** | 0.2458 | **<0.0001** | 0.0282 |  | **0.0442** | **0.0036** |
| miR-148a blood | r | **0.4581** | −0.2396 | **0.6031** | **−0.3856** | **0.5029** | −**0.3423** |  | 0.2514 |
|  | p | **0.0001** | 0.1656 | **<0.0001** | **0.0221** | **<0.0001** | **0.0442** |  | 0.1451 |
| miR-148a ejaculate | r | 0.1597 | **0.3549** | 0.2588 | 0.3069 | **0.0057** | **0.4786** | 0.2514 |  |
|  | p | 0.3595 | **0.0365** | 0.1332 | 0.0730 | **0.9739** | **0.0036** | 0.1451 |  |

**Table S1**. Spearman correlation coefficients and p values for correlation of clinicopathological parameters in PCa patients with miRNA levels; and correlation of miRNAs in blood and seminal plasma. Statistically significant correlation is shown in bold.
